# Assembly and Network Stability of Planktonic Microorganisms under the Influence of Salinity Gradient: an Arctic Case Study from the Lena River Estuary to the Laptev Sea

**DOI:** 10.1128/spectrum.02115-22

**Published:** 2023-02-06

**Authors:** Qian Liu, Yan Li, Hualong Wang, Guipeng Yang, Jinjun Kan, Mengyao Yang, Xiaowen Yu, Cui Guo, Min Wang, Wei Wang, Qingli Zhang, Jiancheng Zhu, Xianyong Zhao, Yong Jiang

**Affiliations:** a College of Marine Life Science & Institute of Evolution and Marine Biodiversity, Ocean University of China, Qingdao, China; b Key Laboratory of Marine Chemistry Theory and Technology, Frontiers Science Center for Deep Ocean Multispheres and Earth System, Ministry of Education, Ocean University of China, Qingdao, China; c Key Lab of Polar Oceanography and Global Ocean Change, Ocean University of China, Qingdao, China; d Yellow Sea Fisheries Research Institute, Chinese Academy of Fishery Sciences, Qingdao, China; e Microbiology Division, Stroud Water Research Center, Avondale, Pennsylvania, USA; Institut Ruder Boskovic

**Keywords:** planktonic microorganisms, prokaryotes, microeukaryotes, community structure, co-occurrence networks, Laptev Sea, Arctic

## Abstract

The diversity and primary productivity in the Arctic ecosystem are rapidly changing due to global warming. Microorganisms play a vital role in biogeochemical cycling. However, the diversity of planktonic microorganism communities in the Laptev Sea, one of the most important marginal seas of the Western Arctic Ocean, have not been studied sufficiently in depth. The diversity and community structure of the planktonic microorganisms in the surface water were investigated at 20 stations on the Lena River flowing into the Laptev Sea. Multivariate statistical analyses demonstrated clear spatial patterns in the α diversity and community structure for microorganisms under different salinity levels. Co-occurrence networks of microbial communities revealed that spatial variation promoted differentiation of the characteristics and stability of microbial networks in the Laptev Sea. Contrary to expectations, abundant taxa were found to not have a large influence on the stability and resilience of microbial interactions in the region. On the contrary, less-abundant taxa were found to have far greater influence. The stability and resilience of the prokaryotic and microeukaryotic networks in the Lena River estuary and the continental shelf provided valuable insights into the impact of freshwater and land inflow disturbances on microbial assemblage. Overall, these results enhance our understanding of the composition of microbial communities and provide insights into how spatial changes of abundant versus rare species alter the nature and stability of microbial networks from the Lena River estuary to the Laptev Sea. In addition, this study explored microbial interactions and their ability to resist future disturbances.

**IMPORTANCE** The regime of the Laptev Sea depends closely on the runoff of the Lena River. Microorganisms are essential components of aquatic food webs and play a significant role in polar ecosystems. In this study, we provided a basic microbial data set as well as new insights into the microbial networks from the Lena River estuary to the Laptev Sea, while exploring their potential to resist future disturbances. A comprehensive and systematic study of the community structure and function of the planktonic microorganisms in the Laptev Sea would greatly enhance our understanding of how polar microbial communities respond to the salinity gradient under climate warming.

## INTRODUCTION

While the earth has warmed by ~0.8°C since the late 19th century, the Arctic region has warmed by 2 to 3°C (1). The rapid warming of the Arctic and the melting of the sea ice have had a major impact on the ecological environment, including changes in the permafrost and the local vegetation or species composition (1). The latest advances in atmospheric science have shown that Arctic amplification, which is the phenomenon of the near-surface air in the Arctic warming much faster than the global average, is associated with the loss of sea ice (2). Arctic amplification may increase the frequency of extreme heat events during the summer at midlatitudes in the Northern Hemisphere. As an Arctic epicontinental sea, the Laptev Sea is of vital interest to understanding how the Arctic responds to climatic and environmental changes. Freshwater input, such as the Lena River, has a substantial influence on the hydrological regimen and plays a crucial role in controlling the temperature and the salinity of the surface water, the extent of sea ice, the terrigenous sediment supply, and the biological processes in the Arctic water (3). The Lena River is one of the largest rivers in the world and is responsible for 20% of the total freshwater discharge into the Laptev Sea (4). The input/discharge of this river leads to the formation of plumes varying in salinity extending 350 km northward and forming a halocline above the Arctic water mass. This results in an extensive salinity gradient (5 to 30 practical salinity units [psu]) in the surface water from the estuary to the continental shelf fault zone (5). In the foreseeable future, as the temperature rises and the permafrost thaws, terrigenous deposits in this region will increase continuously.

Many studies conducted in the Laptev Sea area over the past 2 decades have focused on assessing the impact of the river runoff on the Arctic Ocean ecosystem. Most previous studies have focused on the community composition, distribution, and abundance of benthic fauna (6), zooplankton (7), bacteria and archaea (8), or phytoplankton (9) in environments with various salinities and in various habitats. Some of these studies have described the composition of bacteria and archaea in the Laptev Sea and the effects of changing salinity on the community compositions of meiobenthos and phytoplankton from the Lena River estuary to the Laptev Sea. However, no studies to date have examined the composition and structure of the total planktonic microbial community (including the planktonic prokaryotes and microeukaryotes) and the function of the microbial loop (ML) in the Laptev Sea. The ML concept, proposed by Azam et al. in 1983, emphasizes the ecological importance of the heterotrophic and the autotrophic bacteria specifically, and the microbial organisms more generally, in the food chain (10). Microbial organisms, being essential components of aquatic food webs, play a significant role in polar ecosystems (11, 12). However, our understanding of the structure and function of the planktonic microbial communities in the Laptev Sea of the Siberian Arctic as well as their response to the Arctic system, its hydrography, and the Atlantic Ocean remains unclear (8). A comprehensive and systematic study of the community structure and function of the microorganisms in the Laptev Sea would greatly enhance our understanding of microorganisms in response to climate change.

Previous studies have shown that microbial interactions may be more important than other environmental variables in determining the structure of microbial communities (13, 14). Furthermore, the small size, short generation time, rapid growth, and genetic plasticity of prokaryotes and protists contribute to the complexity of the composition and function of microbial communities. Therefore, exploring the interactions between prokaryotes and microeukaryotes is important for understanding the assembly of microbial communities and their function in the ML of polar oceans. However, the direct study of the various types of interactions between microbial organisms in these ecosystems remains challenging. Network analysis can be used to glean insights into the patterns and potential interactions among microplankton at the community level. Co-occurrence networks can reveal the associations between the organisms and the stability of total communities (15). Co-occurrence networks have been increasingly used to infer microbial interactions (16, 17) not just in soils (18), oceans (13), coastal waters (19), lakes (20), and rivers (21) but also in metabolic modeling (22) and genomic surveys (23). These correlation-based networks provide important insights into the patterns of community interactions, including ecological processes such as cooperation and habitat partitioning, and can be used to determine the mathematical associations among different microbial groups (15, 16). Here, co-occurrence networks were used to study the interaction of the prokaryotic and microeukaryotic communities to further the understanding of the stability and resilience of microbial communities from the Lena River estuary to the Laptev Sea.

This study aimed to increase our understanding of the structure of prokaryotic and microeukaryotic communities, as well as the effects of terrestrial inflow on the assembly of microbial communities and interactions between microbes in the surface waters from the Lena River estuary to the Laptev Sea. Specifically, we (i) characterized the composition and distribution of the planktonic microbial community (including both prokaryotes and microeukaryotes), (ii) evaluated the importance of deterministic and stochastic processes in shaping the community structure and the response of the microbial community to elevated salinity and temperature, and (iii) explored the interactions among the microbes and identified the main factors affecting community structure and microbial interactions in the Laptev Sea.

## RESULTS

### Surface temperature and salinity.

**FIG 1:**
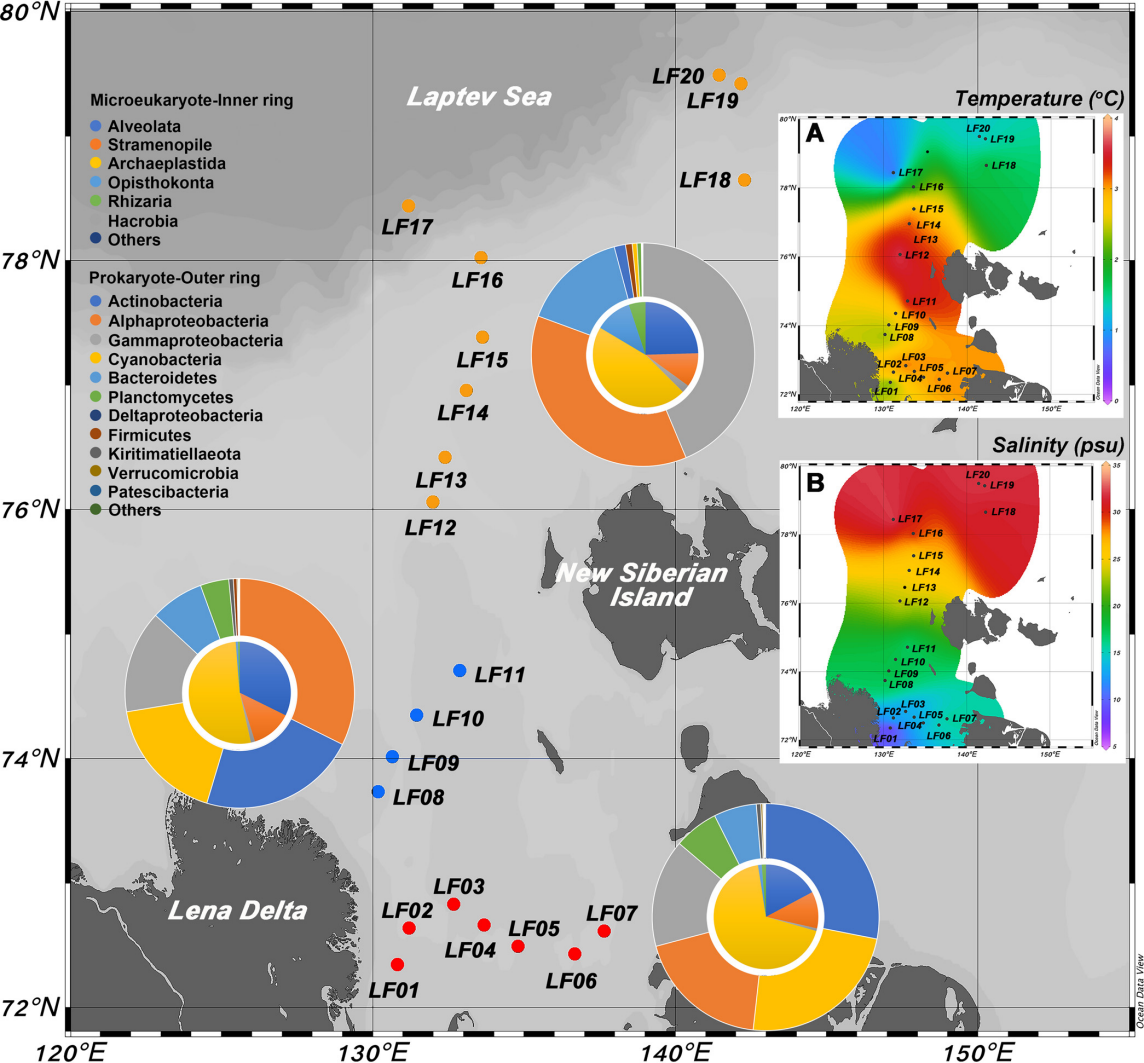
Twenty sampling stations from the Lena River estuary to Laptev Sea during September and October 2018. Pie chart plots indicate the high-ranking taxonomy distribution of the prokaryotic and microeukaryotic community groups. The inner rings represent the relative abundance of microeukaryotes, while the outer rings reflect the relative abundance of prokaryotes. The sampling stations are divided into three groups according to salinity (*P* < 0.05). Red, estuary (LF01-07, the Lena River estuary, low salinity); blue, nearshore (LF08-11, transition region between the Lena River estuary and continental shelf, secondary salinity); yellow, offshore (LF12-20, continental shelf, high salinity). The surface seawater temperature (A) and salinity (B) of 20 sampling stations are shown to the right.

Surface water temperatures were higher in the Lena River estuary (stations LF01 to LF07) and the nearshore (stations LF08 to LF11) than in the continental shelf (LF12 to LF20 [offshore]) (Fig. 1A). The salinity was much higher in the offshore region than in the estuary or the nearshore. The average salinity of the nine sampling stations in the offshore region was 29.17 ± 0.24 psu, and the average salinities of the transition and estuary region were 17.28 ± 0.56 psu and 13.15 ± 0.93 psu, respectively (Fig. 1B).

### Microbial diversity and community composition.

**FIG 2:**
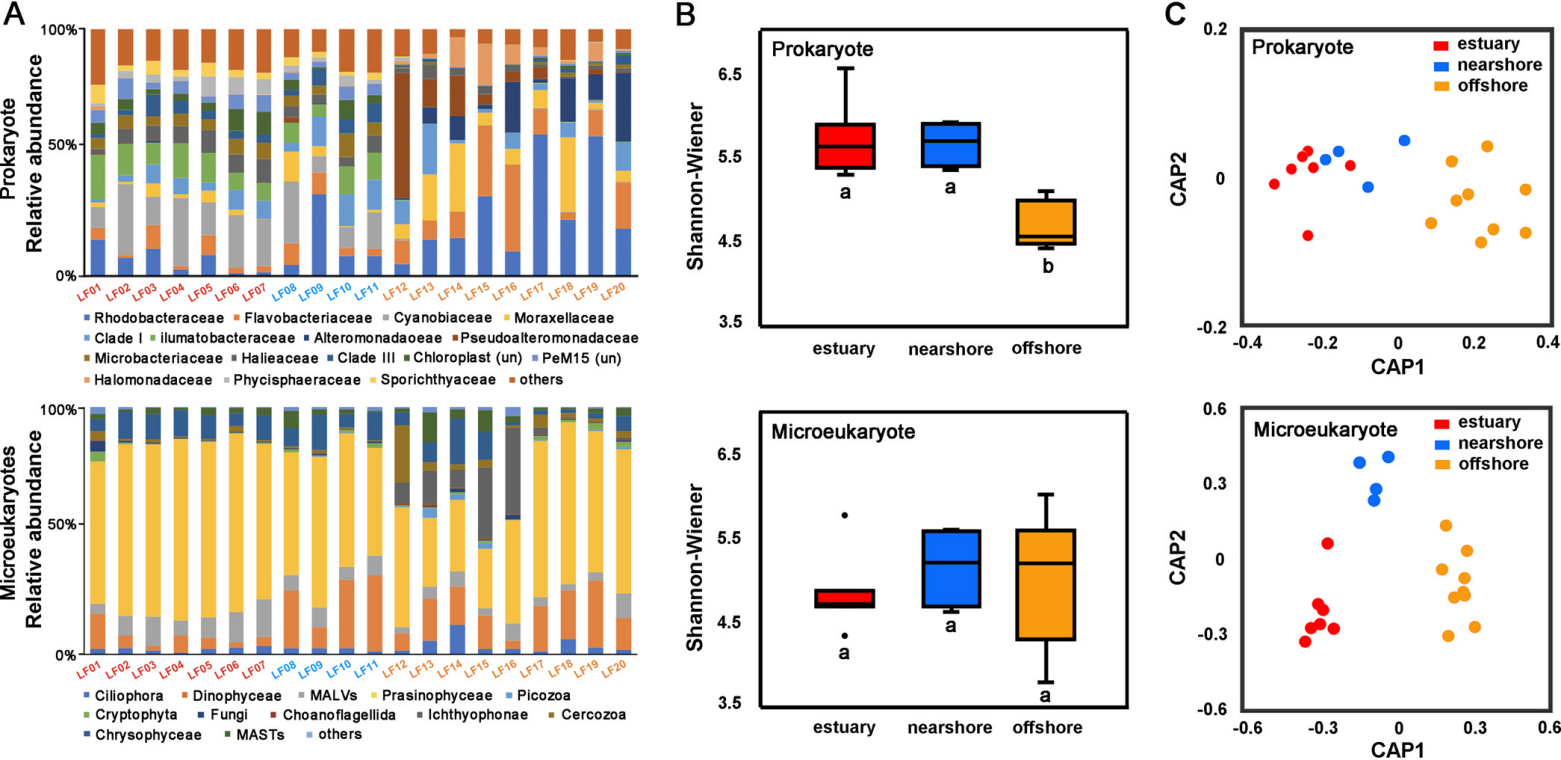
Relative abundance of prokaryotes (top) and microeukaryotes (bottom) at the group and family levels (A). Shannon-Wiener index values of prokaryotes and microeukaryotes are shown in panel B. Values from canonical analysis of principal coordinates based on Bray-Curtis similarities from log-transformed OTU abundance data are shown in panel C.

The taxonomic data in this study comprised a broad spectrum of known bacterial phyla (Fig. 1). The most dominant phylum in all samples was *Proteobacteria*. Numerous sequences affiliated with *Actinobacteria* and *Cyanobacteria* were detected in the estuary and nearshore (Fig. 1); *Bacteroidetes* was the second most abundant phylum in offshore samples (Fig. 1). Community composition at the family level varied across the three sampling regions (Fig. 2A). While both *Cyanobiaceae* and *Ilumatobacteraceae* were highly abundant in the estuary and nearshore samples, they were rarely detected in offshore samples. Although *Moraxellaceae*, *Flavobacteriaceae*, *Alteromonadaceae*, and *Rhodobacteraceae* occurred in all samples from the three regions, their relative abundance was significantly higher in the offshore samples (Fig. 2A). Six supergroups of microeukaryotes, Alveolata, Stramenopiles, Hacrobia, Archaeplastida, Opisthokonta, and Rhizaria, were detected in the samples (Fig. 1). Archaeplastida, Alveolata, and Stramenopiles were the most abundant in all samples across the three sampling regions, whereas Opisthokonta and Rhizaria were more abundant in offshore samples. A total of 24 groups, such as Prasinophyceae, Dinophyceae, Ciliophora, marine alveolates (MALVs), Ichthyophonae, and Chrysophyceae were detected. Prasinophyceae were the most abundant kind of microorganisms in samples from across all three regions. The relative abundance of Chrysophyceae and MALVs was higher in the estuary and the nearshore, whereas the relative abundance of Ichthyophonae was higher in the offshore region than in the other two regions (Fig. 2A).

The Shannon-Wiener diversity of prokaryotes was significantly lower in samples from the offshore region than in those from the estuary and transitional regions (*P* < 0.001). However, the diversity of microeukaryotes was not significantly different among the three regions (*P* > 0.05) (Fig. 2B; see Table S1 at GitHub [https://github.com/qian8341/SUPPLEMENTAL-MATERIAL]). The Bray-Curtis dissimilarity index revealed that β diversity was highest for the prokaryotic and microeukaryotic communities in samples from the offshore region (see Fig. S2 at the GitHub URL above).

Spearman correlation analysis revealed that the most abundant operational taxonomic units (OTUs) were significantly correlated with salinity, and a few of the abundant OTUs were significantly correlated with temperature (*P* < 0.05) (see Fig. S3 at the GitHub URL above). The abundant prokaryotic OTUs were negatively correlated with salinity, and a small number of prokaryotic OTUs were positively correlated with temperature. The abundant microeukaryotic OTUs were positively correlated with salinity but negatively correlated with temperature (Fig. S3). The effect of salinity on the α diversity of prokaryotes was stronger than that of temperature (Table 1).

**TABLE 1:** Spearman correlations of α diversity with salinity and temperature

Parameter	Prokaryotes		Microeukaryotes	
	*R*	*P* ^*a*^	*R*	*P*
Salinity				
Richness	−0.764	**0.001**	0.171	0.427
Shannon-Wiener	−0.737	**0.001**	0.120	0.613
Simpson	−0.606	**0.001**	−0.108	0.650
Pielou’s evenness	−0.669	**0.001**	0.033	0.890
ACE	−0.713	**0.001**	0.099	0.677
Chao 1	−0.737	**0.001**	0.131	0.582
Temp				
Richness	0.134	0.574	0.171	0.250
Shannon-Wiener	−0.017	0.945	−0.301	0.198
Simpson	−0.161	0.498	−0.224	0.301
Pielou’s evenness	−0.077	0.748	−0.232	0.326
ACE	0.110	0.645	−0.248	0.292
Chao 1	0.164	0.490	−0.230	0.329

a Boldface indicates significance at *P* < 0.05.

Discrimination of the community structures was plotted using canonical analysis of the principal coordinates (CAP) on the Bray-Curtis similarity matrices from square root-transformed OTU abundance data (Fig. 2C). The CAP revealed distinct spatial patterns in both prokaryotic and microeukaryotic communities. The microeukaryotic communities significantly differed among the three sampling regions. The first canonical axis (squared canonical correlation [δ_1_^2^] = 0.981) separated microeukaryotic communities in the estuary and nearshore regions from those in the offshore region, and the second canonical axis (δ_2_^2^ = 0.838) separated microeukaryotic communities in the nearshore from communities in the estuary and offshore regions (Fig. 2C). A permutational multivariate analysis of variance (PERMANOVA) revealed significant differences in community structures among the three groups (*P* < 0.005). Unlike microeukaryotes, prokaryotic communities were divided into two groups. The first canonical axis (δ_2_^2^ = 0.978) separated the prokaryotic communities in the offshore region from those in the estuary as well as the nearshore. However, the second canonical axis did not separate the prokaryotic communities in the estuary from those in the nearshore (Fig. 2C). A PERMANOVA also proved that there was a significant difference between the prokaryotic communities in the estuary and nearshore and those in the offshore region (*P* < 0.05). However, there was no significant difference between the prokaryotic communities in the estuary and transitional region (*P* > 0.05).

### Community assembly processes for prokaryotes and eukaryotes.

**FIG 3:**
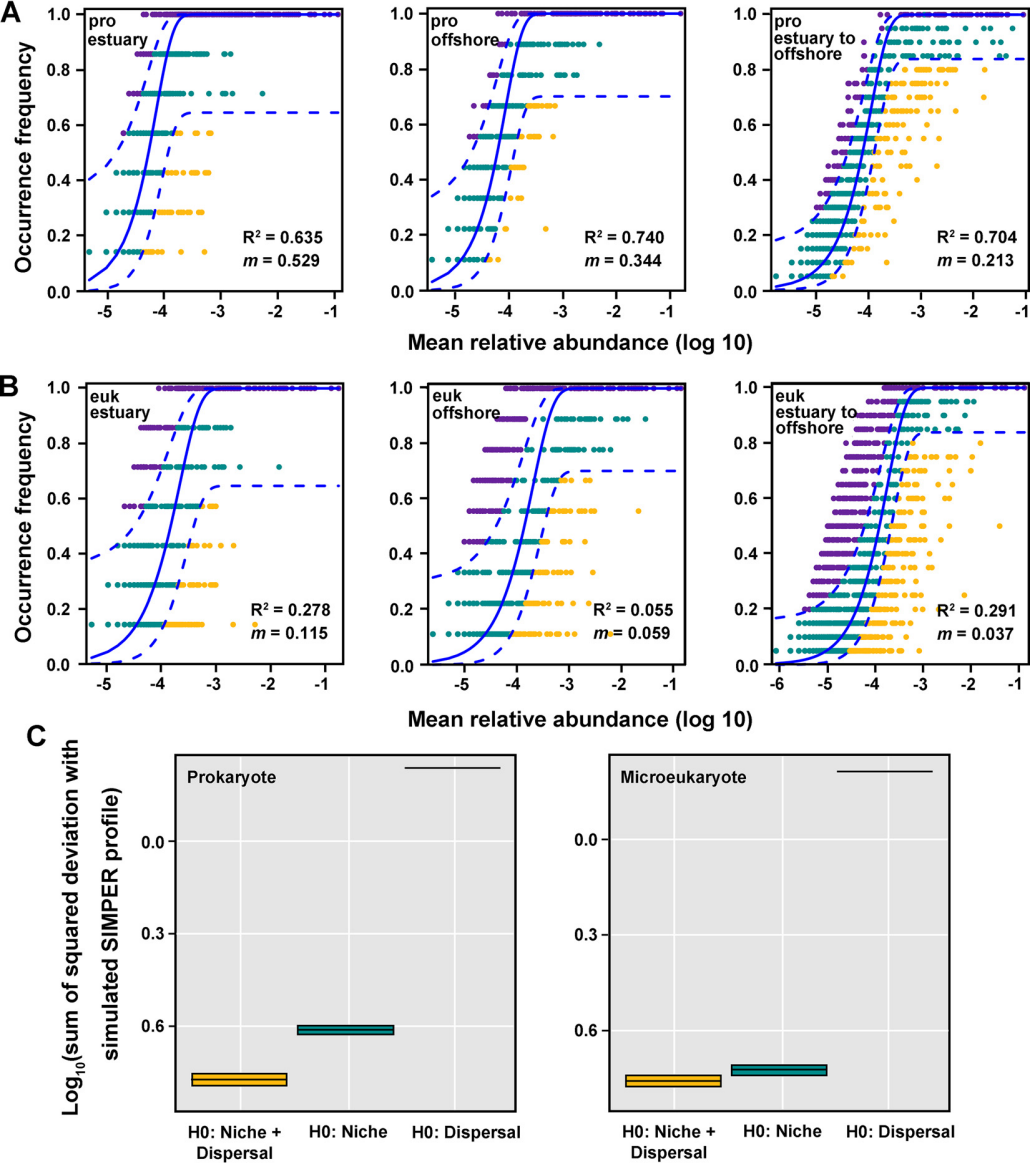
Fit of the neutral community model (NCM) of community assembly. The predicted occurrence frequencies for representing prokaryotes (A) and microeukaryotes (B) from the estuary, offshore, and from the estuary to offshore (including nearshore) are shown in the left, middle, and right panels, respectively. The predicated occurrence frequency is shown as solid blue lines, and dashed blue lines represent 95% confidence intervals around the model prediction. Yellow and purple dots indicate the operational taxonomic units that occur less or more frequently than those given by the model. *m* indicates the metacommunity migration rate, and the *R*^2^ value indicates the fit to this model. (C) E values of the three null models based on the subtraction of the standard effect size.

The Sloan neutral community model (NCM) was used to identify the relative importance of stochastic processes in the assembly of the prokaryotic and microeukaryotic communities (Fig. 3A). The *R*^2^ values (0.635, 0.740, and 0.704 for the estuary, offshore, and the entire study region, respectively) indicated that prokaryotic community assemblages were well described by neutral-based models and that the stochastic processes made important contributions to shaping the assembly of prokaryotic communities in this region. The relative contribution of stochastic processes to the assembly of microeukaryotic communities was lower than it was for prokaryotic communities, as only 27.8, 5.5, and 29.1% of the community variance for the estuary, offshore, and entire study region, respectively, could be attributed to the stochastic processes (Fig. 3B). The immigration rate (*m*) was higher for the prokaryotic community (0.213) than it was for the microeukaryotic community (0.037).

The dispersal-niche continuum index (DNCI) values of prokaryotes and microeukaryotes were −28.354 and −39.394, respectively, which indicated that dispersal assembly was dominant for microbial communities. Models with both sites and species constrained showed the smallest deviation from our data, while those constraining only one of the sites showed the highest deviation (Fig. 3C). That means both niche and dispersal assembly processes affect species distributions.

### Co-occurrence pattern of planktonic microbial communities.

**FIG 4:**
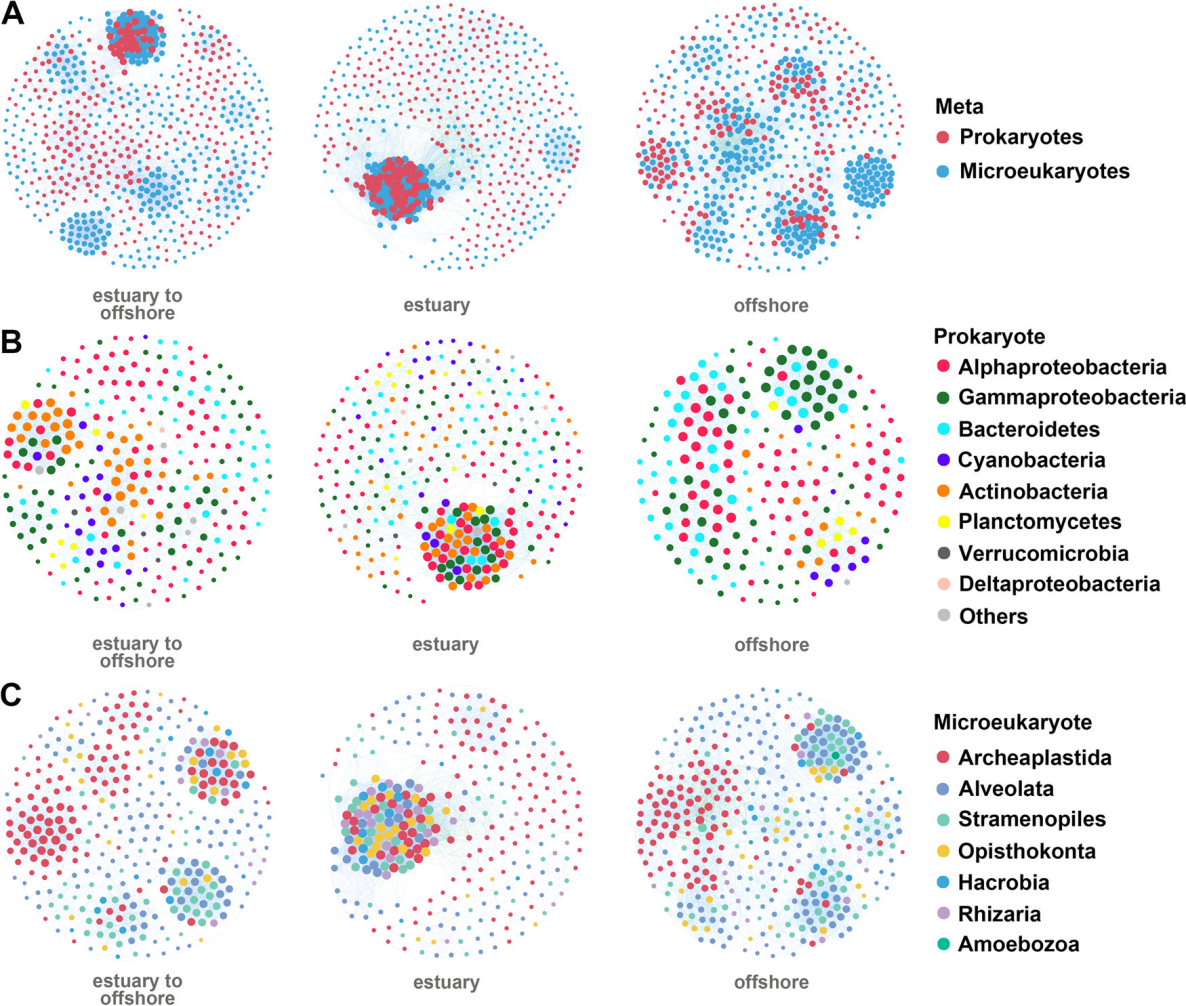
Co-occurrence networks of prokaryotes and microeukaryotes (A), prokaryotes and prokaryotes (B) and eukaryotes and microeukaryotes (C) from the Lena River estuary to the Laptev Sea. The size of each node is proportional to the number of connections (i.e., degree).

Co-occurrence networks were constructed based on the species interaction relationships to determine the co-occurrence patterns of planktonic microbial communities in surface water from the Lena River estuary to the Laptev Sea (Fig. 4A). The results of the planktonic microbial network (meta-network) reflect the interactions between the prokaryotes and the microeukaryotes, consisting of 647 nodes (microeukaryotic and prokaryotic taxa) and 6,743 edges (average degree of 20.844) (see Table S2 at GitHub [https://github.com/qian8341/SUPPLEMENTAL-MATERIAL]). The topological properties of this network are shown in Table S2. The average path length, diameter, clustering coefficient, and modularity of this network were 6.047, 170, 690, and 0.773, respectively. These topological properties indicate the existence of ecological interactions between prokaryotes and microeukaryotes from the Lena River estuary to the Laptev Sea and that ecological niches in this region are shaped by habitat partitioning/sharing.

The aforementioned thresholds were applied to obtain prokaryotic and microeukaryotic networks in the region under consideration (Fig. 4B and C; see Table S3 at the GitHub URL above). Networks of prokaryotes and microeukaryotes and their respective node sizes are shown in Fig. 4B and C. The prokaryotic and microeukaryotic networks were composed of highly connected prokaryotic and microeukaryotic taxa and densely connected groups forming a clustered topology with similar levels of variation. The modularity indices of the prokaryotic and the microeukaryotic networks were greater than 0.4, indicating the presence of modular structures. The numbers of nodes and edges were greater in the microeukaryotic network than in the prokaryotic network (357 nodes and 3,051 edges in the microeukaryotic network versus 273 nodes and 1,659 edges in the prokaryotic network). In addition, the graph density, path lengths, and the clustering coefficient were lower for the prokaryotic community than the microeukaryotic community (Table S2). These findings indicate that the associations between prokaryotes from the Lena River estuary to the Laptev Sea were stronger than those between microeukaryotes.

### Stability of the Laptev Sea microbial networks.

The response capability of network fragmentation (*f*) to remove the top 10 or 20 nodes (i.e., meta-network with 20 nodes removed) with the highest betweenness centrality (how well a node is interacting simultaneously with different compartments of each network, potentially acting as “gatekeepers”), the most relative abundance, and the highest degree (number of connections) was analyzed. Although the number of components and the value of *f* were different from those in the original networks in the case in which nodes with the highest betweenness centrality were removed, few changes were observed when nodes with the highest degree or most relative abundance were removed (Table S2). This suggests that the removal of nodes with the highest betweenness centrality can affect the stability of microbial networks more strongly than the removal of the nodes with the most abundance or the highest degrees. The strong correlations between the betweenness centrality and the degree of node normalization mean that the nodes with high betweenness centrality (gatekeepers) also tended to be of a high degree. Thus, once the gatekeepers were removed, other hub species could replace them and connect different compartments, holding the network together, which could help maintain the stability of the network and enhance its resistance to disturbance. The top 10 or 20 taxa were selected from a ranking based on abundance, degrees, and betweenness centrality, and are listed in Table S4 at the GitHub URL above. The high-betweenness-centrality nodes of the meta-network belonged to Alveolata, Opisthokonta, Stramenopiles, Archaeplastida, *Alphaproteobacteria*, *Kiritimatiellaeota*, *Cyanobacteria*, and *Gammaproteobacteria*. The high-betweenness-centrality nodes of the prokaryotic network belonged to *Actinobacteria*, *Alphaproteobacteria*, *Cyanobacteria*, *Gammaproteobacteria*, and *Kiritimatiellaeota*. Archaeplastida, Alveolata, and Stramenopiles were the nodes with the highest betweenness centrality in the microeukaryotic network. These nodes have a significant effect on the structure and persistence of microorganisms, including the prokaryotic and microeukaryotic communities from the Lena River estuary to the Laptev Sea because they hold the network together. While a few of these nodes were abundant species, the abundance of most of these species was low.

**FIG 5:**
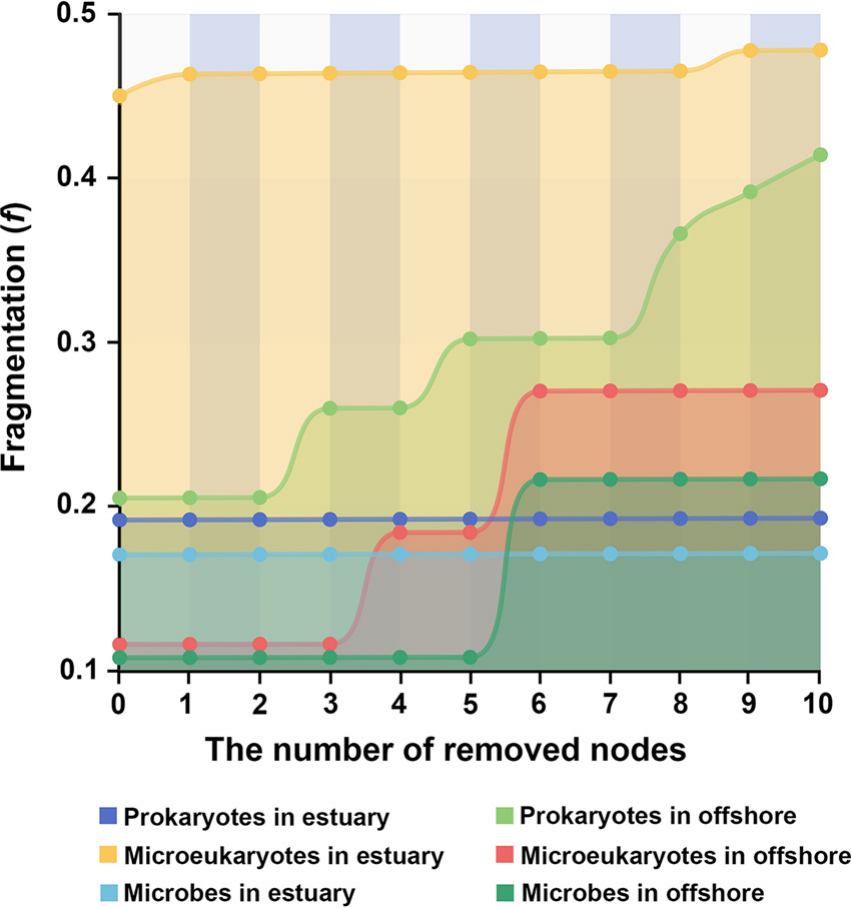
Fragmentations of co-occurrence networks with consecutive removal of 10 or 20 nodes with the highest betweenness centrality.

We further analyzed the stepwise response capability of co-occurrence network fragmentation (*f*) for the removal of the top 10 or 20 nodes with the highest betweenness centrality (Fig. 5; see Table S5 at the GitHub URL). As nodes were removed from prokaryotic networks, *f* began to increase starting with the removal of the second node. While this increase continued as more nodes from the offshore region were removed, no deviations from the initial value were observed when the top 10 nodes from the estuary were removed. In microeukaryotic networks, although the value of *f* changed gradually as nodes from the estuary were removed, it exhibited two abrupt changes in the offshore region. No deviations in the initial value of *f* were observed in meta-networks after the removal of 10 nodes from the estuary. However, a change in the value of *f* was observed when six nodes were removed (Fig. 5; Table S5). These results indicate that the stability of microbial networks varied from the Lena River estuary to the continental shelf in the Laptev Sea, and interactions among the prokaryotes themselves, as well as those between prokaryotes and eukaryotes were more stable and resilient in the estuary. However, the stability of the microeukaryote network was higher in the offshore region than in the estuary. All of the “gatekeepers” in the estuary meta-network were prokaryote taxa, and most of the “gatekeepers” in the offshore meta-network were eukaryotic taxa (Table S5).

### Prediction of bacteria capable of the methane cycle.

**FIG 6:**
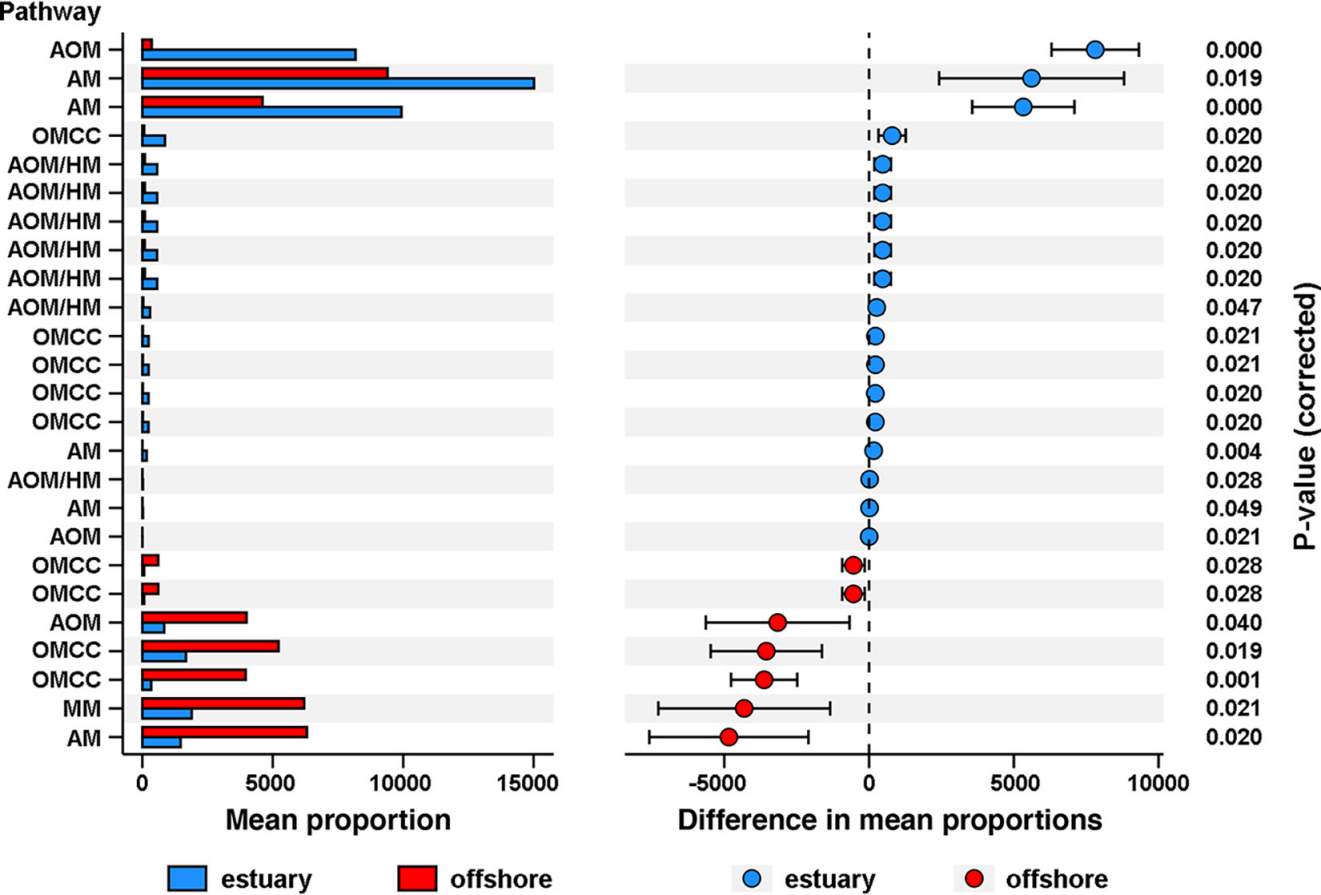
Mean proportions and their differences in predicted functional metagenomes of the methane cycle of bacteria from the Lena River estuary and the Laptev Sea. AOM, anaerobic oxidation of methane; HM, hydrogenotrophic methanogenesis; OMCC, oxidation of methane and C_1_ compounds; AM, aceticlastic methanogenesis; MM, methylotrophic methanogenesis.

The potential metabolic functions of the methane cycle of bacterial communities were predicted by PICRUSt2. Among the 50 genes related to methane cycling functions, the distribution of 25 was shown to be statistically different, with *P* values of <0.05 between the Lena River estuary and the Laptev Sea (Fig. 6). Notably, significant elevations in genes related to the anaerobic oxidation of methane (AOM) and hydrogenotrophic methanogenesis (HM) were observed in samples from the Lena River estuary. On the other hand, a higher incidence of genes related to methylotrophic methanogenesis was detected in samples from the Laptev Sea (Fig. 6).

## DISCUSSION

### Planktonic microbes from the Lena River estuary to the Laptev Sea.

The Laptev Sea has some records of zooplankton or phytoplankton research, but to our knowledge this is the first investigation of prokaryotic and microeukaryotic plankton diversity in the water of the Lena River estuary to the continental shelf using high-throughput sequencing (7, 9). In all, we observed 1,681 prokaryotic OTUs and 1,598 microeukaryotic OTUs, which belong to nine supergroups and 27 phyla, respectively. The results from this study confirmed the overall trends in bacterial taxonomic composition observed in previous studies, with increases in *Alphaproteobacteria* (19.10, 32.35, and 36.93% in the estuary, nearshore, and offshore, respectively) and *Gammaproteobacteria* (15.37, 16.47, and 43.69% in the estuary, nearshore, and offshore, respectively) and decreased in *Actinobacteria* (28.09, 22.28, and 1.63% in the estuary, nearshore, and offshore, respectively) and *Cyanobacteria* (23.67, 17.78, and 0.69% in the estuary, nearshore, and offshore, respectively) with increasing salinity levels (24, 25). However, *Betaproteobacteria* (at 6.09, 7.56, and 15.22% in the estuary, nearshore, and offshore, respectively) were also seen to increase with increasing salinity, which is in contrast to the salinity-driven patterns in the composition of prokaryote communities (24). Like in other high-latitude regions, Archaeplastida, Alveolata, and Stramenopiles were the abundant groups observed (26, 27).

There is a sharp salinity gradient in the surface water from the mouth of the Lena River to the shelf break due to the large amount of runoff (5). The salinity of seawater varied from 6 to 33 psu while traversing from the Lena River estuary to the Laptev Sea in this study. In addition, the Lena River estuary and the adjacent areas are partitioned into actual regions of freshwater influence, as well as a highly complex pattern of temperature and salinity in this region (9). CAP of the 20 samples separated the marine and the brackish prokaryotic communities. Samples in the salinity range 6 to 17 psu (from brackish water in the Lena River estuary and the nearshore) were separated from samples in the salinity range 23 to 33 psu (marine and continental shelf), which indicates the presence of a characteristic brackish prokaryote community in the Lena River estuary. A similar pattern was observed in the Baltic Sea (28). The relative abundance of *Cyanobiaceae* and *Ilumatobacteraceae* is significantly higher in the estuary than on the continental shelf. Here, the salinity in the estuary was markedly lower, and there likely was a concurrent and extensive introduction of freshwater bacteria (29). The environmental conditions, maybe with high terrestrial dissolved organic matter, sorted for species or groups of bacteria that had a metabolic capability to utilize dissolved terrestrial organic matter as their food source (24, 29, 30). The abundance of *Rhodobacteraceae*, gradually increased from the Lena River estuary to the continental shelf, which is consistent with the results of a previous study of prokaryotic communities in a High Arctic fjord (31). CAP separated the microeukaryotic samples from the Lena River estuary, nearshore, and continental shelf, indicating that fewer freshwater and marine eukaryotes can tolerate high levels of salinity. In addition, the distribution of almost all the abundant prokaryotes and microeukaryotes was significantly related to salinity, which underscores the importance of salinity in shaping the structure of microbial communities from the Lena River estuary to the Laptev Sea. This is consistent with the results of previous studies showing that changes in salinity have a significant effect on the structure of plankton communities (32, 33). The composition of eukaryotic communities also varied among the different habitats. Unlike in low-latitude regions, Prasinophyceae was the most abundant group observed. Prasinophyceae is the dominant picophytoeukaryote in polar waters (26), as picoeukaryotes (especially endemic prasinophytes) are thought to be mixotrophic (i.e., osmotrophic and/or phagotrophic), keeping them active throughout the polar night. Such an adaptive strategy might be favorable in polar oceans as opposed to being strictly phototrophic (27). In addition, early season nutrient consumption by phytoplankton blooms under the sea ice and/or at the sea-ice edge in waters can contribute to this overall productivity decline along with a change in the algal community toward smaller cells (34).

### Differences in the assembly of prokaryotic and microeukaryotic communities.

Niche-based theory regards deterministic abiotic factors (environmental factors such as pH, temperature, etc.) and biotic factors (species interactions such as competition, predation, etc.) as the main driver of microbial communities. However, the neutral theory argues that stochastic processes, such as birth, death, migration, speciation, and limited dispersal, shape microbial community structure (35). The NCM is a neutral theory-based process model that allows researchers to quantify the importance of processes that are difficult to observe directly but can have a large influence on microbial communities (i.e., dispersal and ecological drift) (36–40). Furthermore, DNCI offers a way to both quantify and compare the strengths of the main assembly processes across data sets (41). In this study, both NCM and DNCI all revealed that stochastic processes play a key role in shaping assemblages of prokaryotes and microeukaryotes in the water from the Lena River estuary to the Laptev Sea. However, the *R*^2^ values of NCM were much lower for eukaryote communities than for prokaryote communities, suggesting that stochastic processes had a greater contribution to the assembly of prokaryote communities than to the assembly of microeukaryote communities. This result might be explained by the size-plasticity hypothesis (body size effect), which proposes that environmental filtering has a weaker effect on smaller organisms (prokaryotes) than on larger organisms (42). Moreover, the DNCI value and mitigation rate (*m* [NCM]) of the microeukaryotic communities being lower than those of the prokaryotic communities indicated that the dispersal ability of microeukaryotes was lower than that of prokaryotes. Why the slope of the microeukaryote distance-decay pattern was steeper than that for prokaryotes could be partially explained by this (36, 41). A complete understanding of the mechanisms of microbial community assembly in polar water will require additional experimental work, in combination with robust statistical analyses and careful consideration of spatial and temporal axes.

### “Gatekeepers” to planktonic microbial networks.

Co-occurrence network analysis can reveal significant and strong correlations between microbial taxa (43). In this study, planktonic microbial networks consisted of highly interconnected taxa, which formed clustered topologies and thus contained “small-world” properties (nodes are more connected) (44). The abundant microorganisms among the interconnected taxa contribute greatly to the earth’s biogeochemical cycles (45) and are critical for the survival of other organisms (46). However, in this study, we evaluated the response to the removal of the significant nodes with the most relative abundance, highest betweenness centrality, or highest degree (21) to test the stability and resilience of the co-occurrence networks. The most abundant prokaryote or microeukaryote taxa were revealed to not be the “gatekeepers” holding the network together, and hence, they may not be critically important for maintaining the structure or stability of microbial networks. In the present study, most of the gatekeepers were relatively low in abundance and still played crucial roles in the stability, persistence, and resilience of networks. An increasing number of recent studies have revealed the importance of low-abundance taxa in aquatic ecology because they are often more metabolically active than abundant ones and could be the keystone species that regulate the functioning of aquatic environments (47, 48). In addition, the low-abundance taxa have been demonstrated to offer the required gene pool to catalyze the complex degradation processes of organic compounds (49). The capability of the response of network fragmentation to the removal of nodes with the highest betweenness centrality provides important insights into the susceptibility of microbial networks to disturbance (21). This study’s results showed that the loss of potential “gatekeepers” would lead to disproportionate network fragmentation. This is consistent with the results of previous studies of mutualistic networks and food webs showing high susceptibility/fragility following the removal of the specific taxa (50, 51). These removed taxa had the highest betweenness centrality values and were consistently present in major components of the co-occurrence networks. Thus, some of the microeukaryotes and prokaryotes with relatively low abundance play an important role in maintaining the stability of the ML in aquatic environments.

### Stability and resistance of prokaryotic and eukaryotic communities.

Although the stability of microbial communities can be used to draw inferences about the functioning of that ecosystem (52, 53), whether prokaryotic and microeukaryotic co-occurrence networks respond equally to disturbances remains unclear. In particular, the stability and resistance of the prokaryotic and microeukaryotic communities are largely unexplored in the polar oceans. Our co-occurrence networks revealed that interactions between species showed spatial variations similar to those observed in microbial communities. However, the stability and resilience of microeukaryotic and prokaryotic communities varied within the same habitat. Based on the stepwise tests for responsiveness of co-occurrence network fragmentation by the removal of the “gatekeepers,” the stability and resilience of prokaryotic communities were higher in the Lena River estuary than those in the other regions; in contrast, the stability of prokaryotic communities was substantially altered on the continental shelf. There are several possible explanations for this pattern. First, prokaryotes can quickly respond to environmental changes and form new stable communities. They can efficiently utilize phytoplankton exudates, degrade a wide range of substances, including autochthonous and terrestrial dissolved organic matter, rapidly adapt to new growing conditions, and dominate the bacterial community (29). Second, higher biodiversity and a greater number of nodes in the co-occurrence networks suggest that high biodiversity might be able to promote interactions between microbial communities (54). These biotic interactions, including competition, are commonly thought to increase co-occurrence in microbial networks as taxa have access to common resources and are exposed to similar environmental conditions (16, 55). Third, they can probably be driven by a combination of other factors, such as the availability of micronutrients and the composition of dissolved organic matter, that might covary with salinity across the gradient (56). However, the distribution patterns of microeukaryotes are reported to be mainly determined by their salinity preference, or advection, aided by their ability to adapt to brackish conditions rapidly (28).

Unlike prokaryotes, the network interactions of microeukaryotes were more stable in the continental shelf than in the Lena River estuary. The Lena River estuary experiences constant freshwater/oceanic water input and exchange, creating a large salinity gradient from the estuary to the continental shelf. Salinity changes affect not only the biodiversity of eukaryotes and the structure of eukaryotic communities but also the network stability (53). The large salinity gradient had a stronger effect on the properties and stability of the microeukaryotic network. On the continental shelf, which experiences only a weak disturbance from terrestrial inflow due to its distance from the Lena River estuary, the diversity and number of nodes of microeukaryotes were higher, indicating more frequent interactions between species and more complex co-occurrence networks. In the meta-network, which included both microeukaryotes and prokaryotes, the proportion of microeukaryotic nodes was significantly increased in water from the continental shelf. In addition, the node removal test of the meta-networks revealed that prokaryotes play an important role in maintaining the stability of the microbial network in the estuary, whereas microeukaryotes were more important in maintaining the stability of the microbial networks on the continental shelf. This finding is consistent with the results of a previous study examining the structure of microbial taxa in the coastal seawaters of Fildes Peninsula, Antarctica (57). Thus, our findings indicate that microeukaryotes are the key factors in maintaining the stability of microbial networks in the weakly disturbed Laptev Sea.

### Methane cycling of the Lena River estuary versus the Laptev Sea.

In this study, the prediction of bacterial functional profiles focused on key genes related to methane metabolism because our sampling sites were in geographical proximity to the recently discovered field of methane seepage, which significantly contributes to the methane emissions in the Laptev Sea (58). The occurrence of genes predicted for both AOM and HM reactions was significantly higher in samples from the Lena River estuary than in those from the Laptev Sea. HM is the most widespread pathway, and reports suggest that it represents the ancestral form of methane production (59). AOM largely controlled the biological conversion of methane. Water temperature was found to strongly influence the methanogenic pathway, with high temperatures favoring the AOM and HM reactions. The water temperature in the Lena River estuary being higher than the Laptev Sea leads to more genes associated with AOM and HM reactions. To the best of our knowledge, this is the first systematic study to examine microbial interactions (including prokaryotes and microeukaryotes) and their stability in the Laptev Sea. Both univariate and co-occurrence analyses revealed strong correlations between the salinity gradient and the composition of prokaryotic and microeukaryotic communities. The rapid warming of the Arctic region in summer has dramatically altered the environmental conditions in the Laptev Sea, which is increasingly affected by terrestrial inflow from the Lena River. This study contributes a basic microbial data set as well as provides new insights into the microbial networks in the waters from the Lena River estuary to the Laptev Sea and how polar microbial communities respond to the salinity gradients under the influence of global warming.

### Conclusions.

To sum up, our findings provided baseline data that improve our understanding of the spatial variation and potential functions in microbial communities from the Lena River estuary to the Laptev Sea. The spatial patterns and structures in prokaryotic and microeukaryotic communities varied significantly across the transect. The results of the NCM and DNCI indicated that stochastic processes have a dominant effect on the assembly of prokaryotic communities. However, the contribution of stochastic processes to the assembly of microeukaryotic communities was weak by comparison. Changes in salinity caused by terrestrial input might affect the stability and resilience of estuarine microbial interactions from the Lena River estuary to the Laptev Sea. Compared to the dominant taxa, low-abundance groups (“gatekeepers”) had a greater contribution to the topology and stability of networks. Prokaryotes exhibit stronger and more interactive networks in the Lena River estuary than on the continental shelf. Microeukaryotic networks were more stable in the continental shelf. The results of this study demonstrate that biotic interactions play a central role in maintaining the stability of microbial networks and the integrity of the Laptev Sea microbial ecosystem.

## MATERIALS AND METHODS

### Sample collection.

A total of 20 stations from the Lena River estuary to the continental shelf in the Laptev Sea were visited, and surface water samples (2 L each from a depth of about 1 m) were collected during September and October 2018 (Fig. 1). The samples were filtered through 3- and 0.22-μm-pore polycarbonate filters (Millipore, Merck KGaA, Darmstadt, Germany) immediately after collection. These filters were then transferred into 10-mL cryotubes and quickly frozen in liquid nitrogen for further analysis. The temperature and salinity of the surface seawater at each station were recorded with an SBE-19-CTD profiler *in situ*.

### DNA extraction, PCR amplification, and sequencing.

DNA was extracted by the lysozyme-SDS-phenol-chloroform method (26). The V4-V5 region of the 16S rRNA gene was amplified using universal primers 515F (5′-GTGCCAGCMGCCGCGGTAA-3′) and 907R (5′-CCGTCAATTCCTTTGAGTTT-3′) (60), and the V4 region of the 18S rRNA gene was amplified with TAReuk454FWD1F (forward, 5′-CCAGCASCYGCGGTAATTCC-3′) and TAReukREV3R (reverse, 5′-ACTTTCGTTGATYRA-3′) (61) broad microeukaryotic primers. PCR amplification was conducted according to the method described by Liu et al. (57). The concentration of these purified DNA extracts was measured with a Qubit 2.0 fluorometer (Thermo Fisher Scientific, Inc., USA). The purified amplicons were then pooled in an equimolar concentration for paired-end sequencing on an Illumina Miseq PE300 platform. Raw reads in fastq files with low quality (*Q* < 20 or length < 200 bp) were discarded using QIIME (version 1.17) (62). Tags were obtained by merging the paired reads based on their overlaps using COPE (57), after cutting off the sequences of barcodes and primers. Totals of 898,809 (16S) and 1,861,859 (18S) reads were obtained after demultiplexing. Then, UCHIME was used to screen out chimeric sequences and cluster operational taxonomic units (OTUs) at a minimum sequence similarity of 97% (62, 63). Each representative OTU after clustering was compared against the Silva database using a confidence threshold of 70% for taxonomic affiliations. Finally, 1,681 and 1,598 OTUs of bacteria and microeukaryotes, respectively, were obtained (unclassified taxa and metazoans were removed). The rarefaction curves almost approached saturation for each sample (see Fig. S1 in GitHub [https://github.com/qian8341/SUPPLEMENTAL-MATERIAL]).

### Data processing and statistical analysis.

The sampling map, temperature, and salinity diagrams were generated using ODV software (64). The PRIMER (v.7.0) software and R software were used to conduct the multivariate analyses of spatial patterns in the microbial communities (57). The α-diversity indexes (e.g., richness and Shannon Wiener diversity) were generated by the R package “vegan” (65), and analysis of variance (ANOVA) was used to evaluate the differences in indices among the groups. The differences among bacterial communities were visualized by canonical analysis of principal coordinates (CAP) based on the Bray-Curtis dissimilarity matrix (65). PERMANOVA based on 999 permutations was conducted to assess differences among the groups (57). Spearman correlation analyses between log-transformed biotic data (abundant microeukaryotes and prokaryotes and α diversity of microeukaryotes and prokaryotes) and abiotic data (temperature and salinity) were carried out using IBM SPSS (v.22.0). The relationship between the detection frequency and the relative abundance of the prokaryote and microeukaryote taxa was determined using a neutral community model (NCM) to predict the potential importance of stochastic processes in the community assembly (36). To further investigate the relative contribution of stochastic and deterministic factors on community assembly, we calculated the PER-SIMPER and Dispersal Niche Continuum Index (DNCI) (41) using the DNCImper 1.0 package with 999 permutations in R (66).

### Co-occurrence network analysis.

Co-occurrence networks of microbial communities were constructed by Gephi (v.0.9.2) based on Spearman’s rank correlations (*P* < 0.01; *r *>* *0.8 or *r* < −0.8) (57). OTUs of <0.01% in each specific network were removed to reduce the spurious correlation of low-abundance individuals. The topological properties of microbial networks, including component, average clustering coefficient, modularity, network diameter, graph density, and average path length, were calculated in Gephi (v.0.9.2). Network fragmentation (*f*) was calculated as the ratio of the number of disconnected subgraphs (CL) to the overall number of nodes (*N*) in each network, calculated as log (CL)/log(*N*) (21). The average clustering coefficient described how a node was connected with its neighbors on average within the network (67). Modularity quantifies the extent a network can be broken up into smaller components. Modules may represent different niches in microbial networks and have been used to study habitat partitioning/preferences (67). Network diameter, graph density, and average path length were used to briefly describe the density of microbial networks. The average path length was calculated as the average number of steps in the shortest paths between each node.

### Metagenome prediction.

Profiling of predictive microbiota was analyzed by using PICRUSt2 (68). The biom file was uploaded into the online omicstudio terminal (https://www.omicstudio.cn) for preprocessing.

### Data availability.

The sequence data generated in the present study have been deposited in the NCBI Sequence Read Archive (SRA) database under accession no. PRJNA786341 and PRJNA786334.
